# A Case of Inflammation and Suppuration of the Colon, Excited by the Presence of a River Pebble—Its Discharge after Remaining in the Bowels Seven Years, and Recovery of the Patient

**Published:** 1845-10

**Authors:** J. Irwin

**Affiliations:** Nashville


Art. III.—*A Case of Inflammation and Suppuration of the Colon, excited by the presence of a river pebble—its discharge after remaining in the bowels seven years, and recovery of the patient.* Read to the Medical Society of Tennessee, May, 1845. By J. Irwin, M.D.

Miss A. M., aged 16 years, of plethoric habit, was attacked with severe pain in her right side in February, 1842. When I saw her, I found her with a soft and regular pulse, skin natural, tongue clean, and feet cold. In this attack she was confined to bed for two months; the treatment consisted in cupping, leeching, blistering, and fomentations, with purgatives, and the usual remedies, but all afforded no relief. Nothing gave her any relief except morphia; while the system was kept under its influence she complained of no pain, although there still remained great soreness of the side, and this was so great that when she was able to go about the house, she could not bear her clothes to be fastened on her.

In the winter of 1843, she had a similar attack, which I treated with anodynes alone, and she was soon able to be about again, although she still complained of the tenderness of the side. I proposed a seton, but she objected so much that I declined trying it. During the ensuing fall she suffered severely for five or six days with violent acute pain in the same region as formerly, which was relieved as before by morphia, but the soreness still remained. In the following March she was again attacked with acute pain of a throbbing character; her skin and tongue, however, were natural; her pulse soft and regular; bowels costive. I gave her a dose of aloes, rhubarb, and scammony, which operated four times freely, and in one of the discharges there passed from the patient a lump about the size of a nutmeg, which on examination by her mother proved to contain a small irregular pebble, such as is found in rivers and creeks. From this time forward she recovered rapidly, the soreness in her side declined, and she felt nothing of it for six or seven months, when in attempting to learn to weave she strained herself, and was again taken with violent throbbing pain at the same point, which continued for seven or eight days, the side being swollen and very tender on pressure; her stomach became irritable, and in attempting to vomit, she said she felt something break inside, and could feel a fluid run. A few hours after this occurred she had a discharge from the bowels, which consisted chiefly of pure, well-digested pus. The quantity was considerable, and the discharge continued for seven or eight days, when it ceased altogether, and since that time the patient has enjoyed good health and has never complained of her side.

The account given to me by her mother of the introduction of the pebble into her stomach was, that when a child, 7 or 8 years of age, she was in the habit of playing with the pebbles from the river, frequently going to sleep with them in her mouth; in this way, it is supposed, one was accidentally swallowed, and lodging in a fold of the colon remained there for seven or eight years, giving rise to the train of symptoms described.

Nashville, May, 1845.

